# CD47^Low^ Status on CD4 Effectors Is Necessary for the Contraction/Resolution of the Immune Response in Humans and Mice

**DOI:** 10.1371/journal.pone.0041972

**Published:** 2012-08-01

**Authors:** Vu Quang Van, Nobuyasu Baba, Manuel Rubio, Keiko Wakahara, Benoit Panzini, Carole Richard, Genevieve Soucy, Denis Franchimont, Genevieve Fortin, Ana Carolina Martinez Torres, Lauriane Cabon, Santos Susin, Marika Sarfati

**Affiliations:** 1 Immunoregulation Laboratory, Centre Hospitalier de l’Université de Montréal, Research Center (CRCHUM), Notre-Dame Hospital, Montreal, Quebec, Canada; 2 Department of Gastroenterology, Centre Hospitalier de l’Université de Montréal (CHUM), Notre-Dame Hospital, Montreal, Quebec, Canada; 3 Department of Digestive Tract Surgery, Centre Hospitalier de l’Université de Montréal (CHUM), Notre-Dame Hospital, Montreal, Quebec, Canada; 4 Department of Pathology, Centre Hospitalier de l’Université de Montréal (CHUM), Notre-Dame Hospital, Montreal, Quebec, Canada; 5 Department de Gastroenterology, Erasme Hospital, Université Libre de Bruxelles (ULB), Bruxelles, Belgique; 6 Research Institute of McGill University Health Centre, McGill University, Montreal, Quebec, Canada; 7 INSERM U872, Mort Cellulaire Programmée et Physiopathologie des Cellules Tumorales, Equipe 19, Centre de Recherche des Cordeliers, Paris, France; 8 Université Pierre et Marie Curie-Sorbonne Universités, UMRS 872, Paris, France; 9 Université Paris Descartes, Paris, France; National Institute of Infectious Diseases, Japan

## Abstract

How do effector CD4 T cells escape cell death during the contraction of the immune response (IR) remain largely unknown. CD47, through interactions with thrombospondin-1 (TSP-1) and SIRP-α, is implicated in cell death and phagocytosis of malignant cells. Here, we reported a reduction in SIRP-α-Fc binding to effector memory T cells (T_EM_) and *in vitro* TCR-activated human CD4 T cells that was linked to TSP-1/CD47-induced cell death. The reduced SIRP-α-Fc binding (CD47^low^ status) was not detected when CD4 T cells were stained with two anti-CD47 mAbs, which recognize distinct epitopes. In contrast, increased SIRP-α-Fc binding (CD47^high^ status) marked central memory T cells (T_CM_) as well as activated CD4 T cells exposed to IL-2, and correlated with resistance to TSP-1/CD47-mediated killing. Auto-aggressive CD4 effectors, which accumulated in lymph nodes and at mucosal sites of patients with Crohn’s disease, displayed a CD47^high^ status despite a high level of TSP-1 release in colonic tissues. In mice, CD47 (CD47^low^ status) was required on antigen (Ag)-specific CD4 effectors for the contraction of the IR *in vivo*, as significantly lower numbers of Ag-specific CD47^+/+^CD4 T cells were recovered when compared to Ag-specific CD47^−/−^ CD4 T cells. In conclusion, we demonstrate that a transient change in the status of CD47, i.e. from CD47^high^ to CD47^low^, on CD4 effectors regulates the decision-making process that leads to CD47-mediated cell death and contraction of the IR while maintenance of a CD47^high^ status on tissue-destructive CD4 effectors prevents the resolution of the inflammatory response.

## Introduction

During an adaptive immune response (IR), naive T cells responding to Ag proliferate vigorously. While the majority of activated T cells will be killed and eliminated (the contraction phase), effector T cells that have passed this checkpoint will survive and execute their memory T cell differentiation program to generate long-lived memory T cells. Central questions are to determine which cells among proliferating effector T cells will live or die, which cells will be cleared or not, and which factors will dictate these crucial decisions [1]. Although re-expression of IL-7R is a determinant for the survival of effectors that will generate memory T cells [2], [3], no surface molecule has been implicated in the control of cell death and elimination during the contraction phase of the IR. Neither differential Fas expression, nor Fas-induced cell death susceptibility can explain why some effectors die during an acute immune response [4], [5].

CD47, known as integrin-associated protein (IAP), contains a single IgV-like extracellular domain, a multiple membrane-spanning domain (MMS) and a short intracytoplasmic tail, which is devoid of signaling motifs [6]. CD47, considered as a marker of self, is expressed on hematopoietic and non- hematopoietic cells and regulates two key functions implicated in the IR: cell death and cell elimination [7]. CD47 interacts *in cis* with integrins and *in trans* with two ligands, thrombospondin-1 (TSP-1) and signal regulatory protein alpha (SIRP-α). TSP-1 binds two distinct regions on the CD47 IgV loop while it competes with SIRP-α (D1 distal domain) for one of the two CD47 binding sites [8], [9]. SIRP-α/CD47 interaction controls immune cell elimination. CD47 delivers a negative signal through SIRP-α expressed on resident macrophages or dendritic cells (DCs) to inhibit the clearance of intact hematopoietic cells [10]. In this regard, CD47 expression must be transiently up-regulated on circulating wild type hematopoietic stem cells to spare them from clearance during bone marrow exit [11]. TSP-1/CD47 interaction induces the caspase-independent cell death of malignant B and T lymphocytes [7], [12], [13]. TSP-1 is mainly secreted by antigen presenting cells (APCs) and facilitates the clearance of damaged apoptotic cells by APCs [14]. In addition, increased TSP-1 binding facilitates the elimination of aged erythrocytes by SIRP-α^+^ macrophages [15]. We recently reported that CD47 status (SIRP-α Fc binding) is transiently regulated on murine CD4 T cells following *in vivo* immunization. More precisely, CD47^high^ status marked central memory T (T_CM_) CD4 precursors at an early time point of the IR, while CD47^low^ status identified activated CD4 T cells [16].

In the present study, we demonstrated that CD47 expression and more particularly CD47^low^ status on murine activated CD4 T cells, is key for the contraction phase of the IR *in vivo*. In addition, we showed that TCR activation induced a transient change in the CD47 status on human CD4 T cells, i.e. from CD47^high^ to CD47^low^ to CD47^high^, which was linked to TSP-1/CD47-mediated cell death *in vitro*. Importantly, CD47^low^ status was maintained on CD4 effectors cells in inflamed lymphoid and mucosal tissues of patients with Crohn’s disease (CD). We thus propose that CD47/SIRP-α/TSP-1 axis is involved in the resolution of the inflammatory response.

## Results

### 1. CD47 Status is Differentially Regulated on TCR- Activated Human CD4 T Cell Subsets

**Figure 1:**
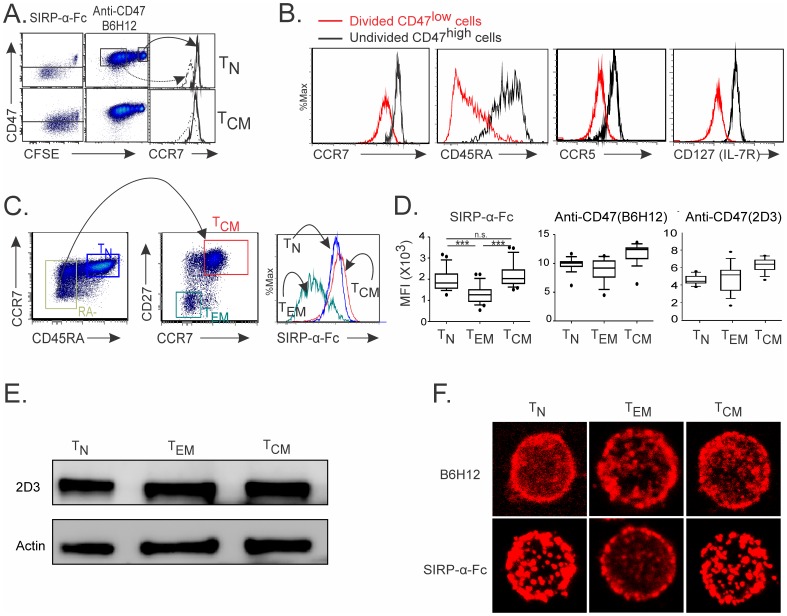
CD47 status is differentially regulated on TCR- activated human CD4 T cell subsets. (**A–B**) CFSE-labeled T_N_ and T_CM_ cells were stimulated with immobilized anti-CD3 and soluble anti-CD28 mAbs for 6 days. (A) CD47 (using human SIRP-α-Fc protein or anti-CD47 mAb, clone B6H12) and CCR7 expression was analyzed by flow cytometry. (B) Phenotype of divided CD47^low^ and undivided CD47^high^ cells at day 6 of T_N_ cultures. (**C**) Strategies to examine CD47 expression on *ex vivo* isolated human T cells gated on CD4^+^ T cells. (**D**) CD47 expression on CD4 T cell subsets using SIRP-α-Fc and anti-CD47 antibodies (B6H12 and 2D3). The mean ± standard deviation (SD) for 16 donors is shown (Anova test: ***p<0.0001). (**E**) Western blot analysis for CD47 protein on whole-cell lysates using 2D3 mAb. (**F**) Confocal immunofluorescence of CD47 using SIRP-α-Fc or anti-CD47 (B6H12) antibodies. (**A–C; E and F**) Data are representative of 3 to 6 independent experiments.

We first investigated the modulation of CD47 expression on *in vitro* activated human CD4 T cell subsets. To this end, we thought to use a SIRP-α-Fc fusion protein and two anti-CD47 monoclonal antibodies (mAbs) that identify different CD47 conformations [15], [17], [18], [19], [20] and/or distinct CD47 epitopes [21]. Hence, B6H12 mAb and SIRP-α-Fc compete for a similar CD47 binding site since B6H12 but not 2D3 inhibits SIRP-α-Fc binding to CD47 [22]. We showed that CD47 expression, as detected by SIRP-α-Fc binding, decreased on a majority of divided naïve CD4 T cells (T_N_; CD45RA^+^CCR7^+^) following stimulation with anti-CD3 and anti-CD28 mAbs (Fig. 1A). The reduced CD47 expression was not observed when activated CD4 T cells were stained with B6H12 anti-CD47 mAb. Thus, decreased SIRP-α-Fc binding to CD47 on activated T_N_ cells was hereafter referred to as CD47^low^ status when compared to SIRP-α-Fc binding to CD47 on undivided T_N_ cells as well as on 50% of activated central memory (T_CM_; CD45RA^-^CCR7^+^CD27^+^) T cells hereafter referred to as CD47^high^ status (Fig. 1A). Divided CD47^low^ CD4 T cells displayed an effector phenotype (CCR7^low^) when compared to undivided CD47^high^ CD4 T cells (Fig. 1B).

Further studies demonstrated that CD47 status was differentially modulated in *ex vivo* isolated circulating human CD4 T cell subsets (Fig. 1C). Effector memory (T_EM;_ CD45RA^−^CCR7^−^CD27^−^) T cells, which represent chronically activated T cells by repeated exposure to Ag in the peripheral blood of healthy individuals, displayed a CD47^low^ status when compared to CD47^high^ T_N_ and T_CM_ T cells (Fig. 1D). Transitional memory (T_TM_, CD45RA^−^CCR7^−^CD27^+^) and terminally differentiated (T_TD_, CD45RA^+^CCR7^−^CD27^−^) cells were detected as CD47^low^ cells. Alike *in vitro* TCR-activated CD4 T cells, T_N_, T_EM_ and T_CM_ expressed similar levels of CD47 expression when they were stained with B6H12 and 2D3 anti-CD47 mAbs, suggesting a change in the conformation rather than in the amount of CD47 protein. Indeed, western blot analysis showed that the three circulating CD4 T cells subsets possessed similar CD47 protein content (Fig. 1E). We next investigated whether differences seen in SIRP-α- Fc binding to CD47 may reflect a differential distribution of CD47 on the cell surface of T_N_, T_EM_ and T_CM_. As depicted by confocal microscopy, SIRP-α- Fc staining revealed a homogenous distribution of CD47 molecules on the cell surface of T_N_ and T_CM_ while a distinct and patchy redistribution was observed on T_EM_ (Fig. 1F). In contrast, no characteristic CD47 distribution was found between T_N_, T_EM_ and T_CM_ using B6H12 mAb. Additionally, SIRP-α-Fc failed to bind CD47 with a truncated transmembrane domain in a modified human T cell line (*Fig. S1*). We propose that TCR activation elicits a post transcriptional/translational modification of the CD47 molecule that dictates its ability to bind SIRP-α-Fc but not B6H12 or 2D3 mAbs. These data strongly suggested that CD47 status on T_EM_ and T_CM_ subsets reflect different CD47 protein conformations, as these T cells possessed similar protein content.

### 2. IL-2 Induces a CD47^high^ Status on Human TCR-activated CD47^low^ CD4 T Cells

**Figure 2:**
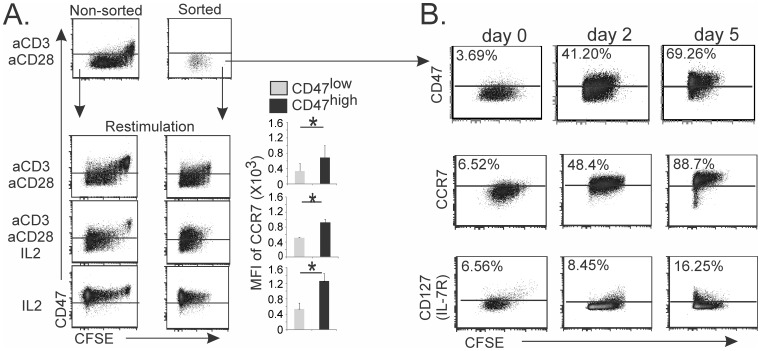
IL-2 induces CD47^high^ status on TCR-activated human CD47^low^ CD4 T cells. (**A**) CFSE-labeled T_N_ cells (left panels) were activated with immobilized anti-CD3 and soluble anti-CD28 mAbs for 6 days. Unfractionated activated T cells or FACS sorted CD47^low^ T cells (middle panels) were restimulated for 5 days as indicated. CD47 expression in relation to cell division (CFSE cell dilution) and CCR7 expression in relation to CD47 status. (**B**) T_N_ were activated as in A and FACS sorted CD47^low^ (day 6) T cells were restimulated in presence of IL-2. Kinetics of CD47 (SIRP-α-Fc protein), CCR7 and CD127 expression is shown. (**A–B**) Data are representative of at least 4 to 6 independent experiments. Right panel A shows the mean ± standard deviation (SD) for 5 independent experiments. Student *t* test: *p<0.05.

Several cytokines that signal through receptors sharing the common γ chain (γc) are critical for peripheral homeostasis and the generation of memory T cells [23], [24]. We found that a large proportion of TCR-activated naive CD4 T cells regained a CD47^high^ status when these cells were cultured in the presence of IL-2, with or without CD3 restimulation and co-stimulation (Fig. 2A). This suggested that CFSE^low^CD47^high^ activated T_CM_ cells arose from CD47^low^ T cells. To rule out the possibility that CD47^high^ T cells originated from a few CD47^high^ T cells, which had proliferated and never modified their CD47 status, CD47^low^ CD4 T cells were purified at the end of T cell primary cultures (day 6) and then were re-stimulated. We demonstrated that, indeed, IL-2 induced the re-establishment of a CD47^high^ and central memory (CCR7^high^) phenotype in FACS sorted purified TCR-activated CD47^low^ CD4 T cells (Fig. 2B). The appearance of CD47^high^ effectors preceded that of CD127 positive cells (Fig. 2C). Thus, human CD4 T cells transiently modulate their CD47 status in response to polyclonal activation, and T_CM_ phenotype is associated with the reacquisition of a CD47^high^ status.

### 3. CD47^low^ Status is Linked to TSP-1-induced Cell Death Susceptibility

**Figure 3:**
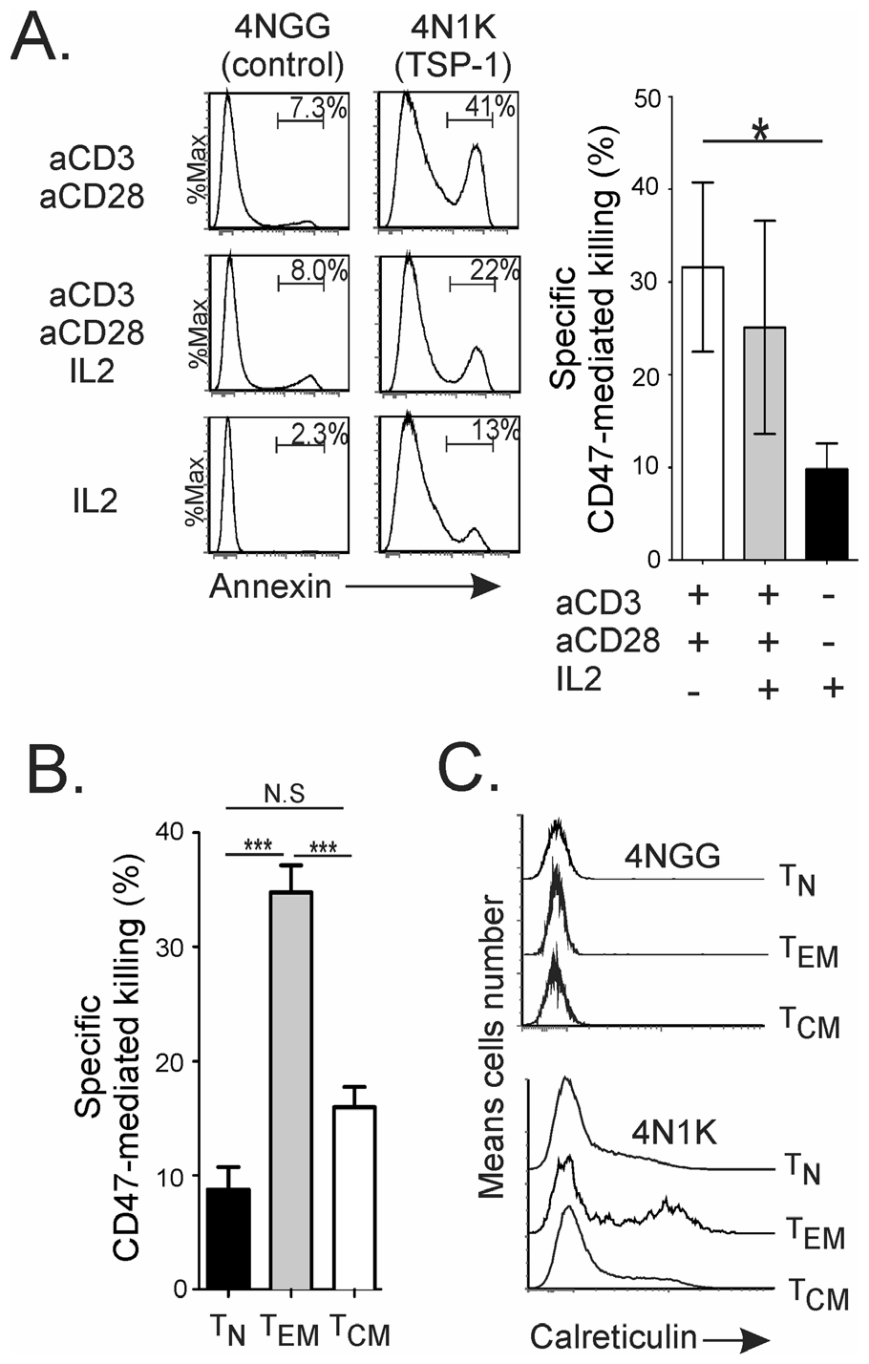
A CD47^low^ status is linked to TSP-1-induced cell death susceptibility. (**A–B**) Specific CD47-mediated killing was performed using 4N1K (TSP-1) or 4NGG (control) peptide on *in vitro* restimulated FACS sorted activated CD47^low^ T cells as in Figure 2 (A) and *ex vivo* isolated CD4 T cell subsets (B). (**A–B**) The mean ± standard deviation (SD) for 5 independent experiments. Student *t* test: *p<0.05, ***p<0.0001. (**C**) Calreticulin expression is shown after specific CD47-mediated killing as in B. Data are representative of 4 independent experiments.

We next explored whether the transient modulation of CD47 status seen on CD4 T cells might be linked to functional consequences such as T cell death, which occurs during the resolution of the IR. Ligation of CD47 by 4N1K, a peptide that corresponds to the CD47-binding C-terminal domain of TSP-1, kills malignant B cells and T cell lines through a caspase and Fas-independent pathway [13], [25], [26], [27]. We therefore assessed the TSP-1/CD47-mediated cell death of human CD4 T cells in relation to their CD47^low^ or CD47^high^ status. TCR-activated CD47^low^ T cells were susceptible to 4N1K-induced cell death (Fig. 3A). However, they became resistant when they were cultured in the presence of IL-2 and reacquired a CD47^high^ status, linking a change in the CD47 status to susceptibility to TSP-induced cell death. Specific CD47-mediated killing was demonstrated in T_EM_, while T_N_ and a large fraction of T_CM_ were largely protected from 4N1K-induced cell death (Fig. 3B), corroborating our *in vitro* findings with TCR-activated T cells. We next asked whether differential CD47 status on CD4 T cell subsets correlated with the switching on of one common “eat me” signal, i.e. calreticulin, which favors cell elimination when CD47/SIRP-α interactions are interrupted [28]. Expression of calreticulin was not detected on viable T_N_, T_EM_, or T_CM,_ although it was significantly induced on T_EM_ killed by 4N1K (Fig. 3C). We propose that killing of CD47^low^ T cells occurs upstream of cell clearance, with the latter being mediated by up-regulation of “eat me” signals combined with the interruption of SIRP-α/CD47 interactions.

### 4. Chronically Activated T Cells Display a CD47^high^ Status in Lymphoid and Intestinal Tissues of Patients with Crohn’s Disease

**Figure 4:**
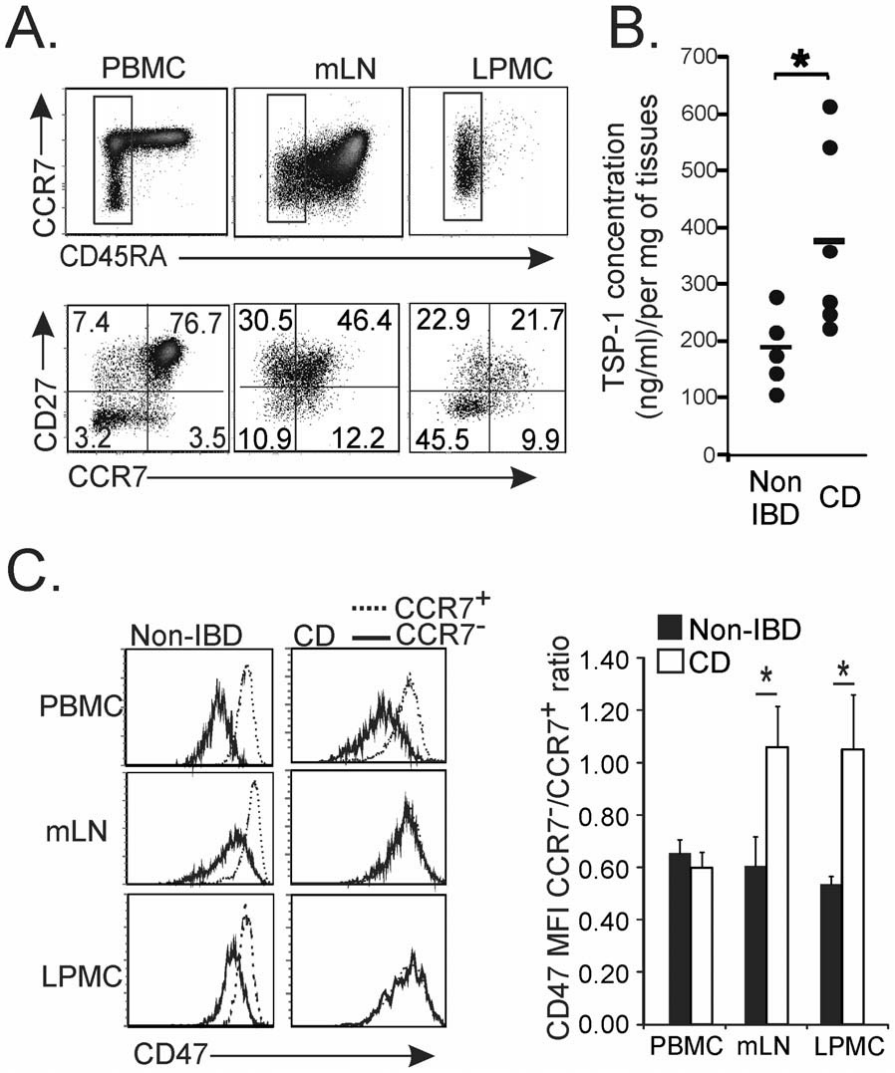
CD4 effectors display a CD47^high^ status in lymph nodes and lamina propria of patients with Crohn’s disease. (**A**) CD4 T cell subsets were examined in PBMC, mLNs and LPMC of patients with Crohn’s disease. (**B**) TSP-1 concentration in human colon biopsies. (**C**) CD47 expression (SIRP-α-Fc protein) after gating on memory (CCR7^+^) and effector (CCR7^−^) CD45RA^−^CD4^+^ T cells in PBMC, mLNs and LPMC. Data are representative of 4 to 6 independent experiments. The CD47 Mean Fluorescence Intensity (MFI) CCR7^−^T/CCR7^+^ T cell ratio was calculated for patients with CD and unrelated IBD patients. (**A and C**) The mean ± standard deviation (SD) for 5 to 6 independent experiments. Student *t* test was performed: *p<0.05.

Survival of auto-aggressive T cells in tissues prevents the resolution of the inflammatory response and perpetuates disease in patients with inflammatory bowel disease (IBD) [29]. More specifically, lamina propria T cells appear resistant to cell death in Crohn’s disease (CD). We therefore asked whether escape to cell death of mucosal CD4 T cells correlated with their CD47 status in CD patients. To this end, we examined binding of SIRP-α-Fc to CD47 on CD4 T cells in blood, mesenteric lymph nodes (mLNs) and intestinal lamina propria mononuclear cells (LPMC) of CD patients. As expected, the frequency of CD4 effectors (CD45RA^−^CD27^+/−^CCR7^−^) was increased in mLNs and LPMC when compared to PBMC, whereas that of T_CM_ (CD45RA^−^CD27^+^CCR7^+^) was reduced accordingly (Fig. 4A). Despite abundant TSP-1 release in inflamed colonic CD tissues (Fig. 4B), both CCR7^+^ and CCR7^−^ CD4 T cell subsets that infiltrated mLNs and inflamed gut tissues expressed a CD47^high^ status (Fig. 4C) that could explain the maintenance of auto-aggressive T cells. However, as in healthy donors (Fig. 1), circulating T_EM_ and T_CM_ displayed a CD47^low^ and CD47^high^ status, respectively in patients with CD or an unrelated intestinal disorder (non IBD)(Fig. 4). To verify that absence of differential CD47 status on CCR7^+^ and CCR7^−^ memory T cells was not a property of T cells that were recruited to peripheral tissues, we also examined mLNs and LPMC of patients with non IBD. As depicted in the same figure, CCR7^−^ effectors displayed a CD47^low^ status in mLNs and colons of non IBD donors as reflected by the ratio of CD47 mean fluorescence intensity (MFI) between CCR7^−^ and CCR7^+^ T cells. These data suggest that CD4 effectors maintain a CD47^high^ status in inflamed colons, which confers them a resistance to TSP-1-mediated cell death in tissues and favors their accumulation.

### 6. Expression of CD47 is Required on Murine Ag-specific CD4 T Cells for the Contraction Phase *in vivo*

**Figure 5:**
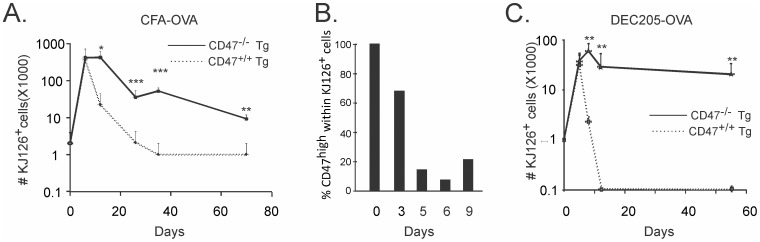
CD47 on CD4 T cells regulates the contraction of the immune response *in vivo*. One day after adoptive transfer of CD47^+/+^ or CD47^−/−^ Tg T cells isolated from DO11.10 mice into CD47^−/−^ BALB/c hosts, mice were immunized s.c. with CFA-OVA or DEC205-OVA. (**A and C**) Kinetic of the recovery of viable Tg T cells is shown. (**B**) CD47 status (SIRP-α-Fc protein) gated on CD4 KJ126^+^(Tg) T cells post immunization. Data are representative of 4 to 6 independent experiments, student *t* test was performed on 8 to 12 mice. *p<0.05, ***p<0.001.

We recently reported that CD47^low^ status is observed on activated murine CD4 T cells *in vivo,* independently of the route and the methods of immunization [16]. We hypothesized, here, that CD47^low^ status and CD47-mediated cell death are involved in the crash of the IR, while the reestablishment of a CD47^high^ status might offer an advantage to pre-committed T_CM_ cells to escape cell death and elimination. Indeed, CD47^high^ status marked T_CM_ precursors at an early time point of IR [16]. We therefore determined whether CD47 expression and/or CD47 status on murine CD4 T cells had an impact on the contraction phase of the IR *in vivo*. CD47 is implicated in cell elimination [10]. Hence, viable CD47^−/−^ Tg T cells are readily cleared from wild type hosts, whereas they can be adoptively transferred into CD47-deficient hosts without being cleared [30], [31]. Here, CD47^−/−^ hosts were passively transferred with Tg CD47^−/−^ or CD47^+/+^ CD4 T cells and immunized subcutaneously with CFA/OVA. Kinetics revealed that proliferation of Tg CD47^+/+^ T cells occurred at an early time point followed by cell contraction (Fig. 5A). The latter correlated with a change to a CD47^low^ status (Fig. 5B). Tg CD47^+/+^ T cells also proliferated, albeit at a lower rate, in DEC205-OVA when compared to CFA/OVA immunized mice before their elimination from hosts (Fig. 5C). Although the recovery of Tg CD47^+/+^ and CD47^−/−^ CD4 T cells was similar until day 9, the absence of CD47 on CD4 T cells significantly increased the yield of Ag-specific T cells at later time points in both immunogenic (CFA/OVA) and tolerogenic (DEC205-OVA) responses (Fig. 5). Tg CD47^−/−^ T cells were still retraced 70 days after immunization. Therefore, we demonstrate that CD47 expression, and specifically a CD47^low^ status, is required on CD4 T cells for the contraction phase during an acute immune response.

## Discussion

The pathway to memory T cell generation can be divided into 3 sequential and critical steps during an acute immune response: 1) resistance to massive cell death, 2) prevention of viable cell elimination, and 3) cell survival. CD47 is implicated in the two first steps [7]. We propose here that a change to a CD47^low^ status is key to determine the cell’s decision to die while the commitment to cell clearance occurs as a downstream event. The CD47^low^ status was detected by staining CD4 T cells with a SIRP-α-Fc fusion protein, although it was not observed using two anti-CD47 mAbs that recognize distinct epitopes. Combined with flow cytometry data, the confocal microscopy and Western blot analysis suggest that CD47 status is linked to a post-translational modification and/or cell surface redistribution rather than to differences in the amounts of CD47 protein expression. A change in the CD47 conformation has been reported in several earlier studies. In fact, binding of B6H12, 2D3 mAbs and SIRP-α-Fc to CD47 is regulated by several factors such as temperature, the cell type on which CD47 is expressed and the presence of cholesterol [17], [32]. For example, affinity of B6H12 and 2D3 mAb for CD47 is higher when monocytes but not RBC, are incubated at 37C instead of 0C. In addition, CD47 displays a different conformation on sickle RBC when compared to normal RBC [18]. 2D3 binds with greater affinity than B6H12 mAb to CD47 on sickle [18] and aged erythrocytes [15], which results in adhesion to TSP-1 under ﬂow and static conditions. A loss of SIRP-α-Fc but not B6H12 and 2D3 binding, thus acquisition of CD47^low^ status, can be artificially induced either by replacing the transmembrane region of CD47 by CD7, by cholesterol removal, or by a double cysteine mutation that disrupts the S-S disulfide bridge between the transmembrane and extracellular CD47 domains [19], [20]. Nonetheless, the precise molecular mechanism behind the physiologic conformational modification of CD47 remains to be elucidated. Reduced or enhanced binding to SIRP-α-Fc might result from either CD47 association with other surface molecules, since CD47 was originally identified as an integrin-associated molecule [33], CD47 redistribution at the membrane [28], and/or a modified glycosylation pattern [22].

In the present study, we investigated the functional consequences provoked by the change in CD47 status on CD4 T cells. Upon activation, human CD4 T cells transiently displayed a CD47^low^ status and become sensitive to CD47-mediated cell death by TSP-1. This may represent one mechanism involved in the contraction of the IR, as well as in the resolution of the inflammatory response. We here showed that the absence of CD47 on murine Ag-specific T cells significantly impaired the contraction of the IR *in vivo*, demonstrating that the presence of CD47, and more particularly a CD47^low^ status, was necessary for this process to occur. Furthermore, a transient change of CD47^low^ status on CD4 T cells is required to mediate TSP-1-induced cell death *in vitro* in humans. IL-2 induced a re-expression of CD47^high^ status on human TCR-activated CD4 T cells. T cells themselves represent a source of IL-2 and TSP-1 and CD3 stimulation leads to an increase in the availability of TSP-1 on the cell surface of recently activated T cells [34]. CD47 ligation inhibits early T cell activation, IL-2 production, and CD25 expression [35]. The latter is transiently expressed on activated CD4 T cells *in vivo,* and CD4^+^CD25^−/−^ or IL-2^−/−^ effector T cells survive very poorly and generate low numbers of memory T cells in non lymphopenic naive mice [36]. TSP-1 and SIRP-α bind CD47 IgV loop [37] and TSP-1 can inhibit SIRP-α-Fc binding to CD47 expressing-Jurkat cells [38]. We therefore postulate that the reestablishment of a CD47^high^ phenotype on T_CM_ and re-encounter with SIRP-α^+^ myeloid cells (macrophages or DCs) might offer an advantage to avoid TSP-1-induced cell death whereas CD47^low^ status promotes TSP-1 binding that favors cell death and elimination. A direct interaction between CD47^low^ effectors and SIRP-α^+^ DCs may also induce IL-2 secretion by T cells. Rebres et al. have demonstrated that SIRP-α-Fc ligation synergizes with CD3 for T cell activation and induces PKC θ translocation, resulting in IL-2 production by T cells [19]. DCs, through autocrine secretion of IL-2, trans-present IL-2 to T cells for optimal clonal expansion and effector function [39]. Thus, in addition to T cell-derived IL-2, DCs also could reverse a CD47^low^ to a CD47^high^ status. We showed here that a CD47^high^ status was maintained on CD4 effectors in inflamed CD tissues. This suggests that auto-aggressive T cells that contribute to tissue destruction, might possess a deregulation in the conformational change process of CD47 which is revealed by an increase in SIRP-α-Fc binding. CD47^high^ status confers resistance to TSP-1-induced killing to CD4 tissue effectors that accumulate in tissues, as we observed abundant TSP-1 release in CD tissues. In that regard, we recently reported that CD47^high^ status on CD4 effectors identifies functional long-lived memory T cell progenitors [16]. Therefore, maintenance of a CD47^high^ status in pathology may be deleterious to the host and perpetuate chronic inflammatory response.

How effector T cell death is regulated during the contraction phase is not fully understood. For many years, the Fas death receptor was considered to be the only T cell surface molecule implicated in the contraction phase of the IR. Fas-mediated signaling leads to activation-induced caspase-dependent apoptosis of TCR-expanded T cells [40], [41]. Mice lacking functional genes for Fas or its ligand (FasL), show uncontrolled lympho-proliferation and developed autoimmunity [42], [43]. CD47 has been linked to Fas [44]. Yet, CD47^−/−^ mice do not display lympho-proliferative disorders as seen in Fas-deficient mice [45]. Fas signaling, like CD47, kills T_EM_ cells and spares T_CM_ as well as T_N_ cells. However, unlike differential CD47 status, Fas expression is similar on T_EM_ and T_CM_ cell subsets [46]. CD47 augments Fas-mediated apoptosis, but CD47-initiated signaling is not required to enable Fas killing. This process is unidirectional since Fas is not necessary for CD47-mediated killing [44]. We thus propose that CD47, rather than Fas-mediated cell death, plays a key role in the dampening of an acute response. We showed here an increased yield of Ag-specific CD47^−/−^ T cells in CD47^−/−^ hosts while Ag-specific CD47^+/+^ T cell were barely detectable 70 days after primary immunization. In fact, the role of Fas in contraction phase has been challenged by Alexander *et al*, who showed that the elimination of effector T cells is completely independent of caspase activation. Administration of 11 different regimens of a pan-caspase inhibitor benzyloxycarbonyl-Val-Ala-Asp (OMe)-ﬂuoromethylketone (zVAD) *in vivo* showed no significant impact on effector or memory CD8 or CD4 T cell development [4]. Neither the activation of caspases nor that of pro-apoptotic members of the Bcl-2 family, such as Bax, Bak or Bim, or the release of apoptogenic proteins AIF (apoptosis-inducing factor), cytochrome c, endonuclease G (EndoG), Omi/HtrA2 and Smac/DIABLO from mitochondria tocytosol is observed in CD47-mediated cell death [12], [25]. Instead, the molecular pathway of CD47-caspase–independent cell death involves Drp1 translocation from the cytosol to the mitochondria, a process controlled by chymotrypsin-like serine proteases [25]. Once inside the mitochondria, Drp1 provokes an impairment of the mitochondrial electron transport chain, resulting in dissipation of mitochondrial transmembrane potential, reactive oxygen species generation, and a drop in ATP levels. However, a physical interaction between CD47 and the proapoptotic Bcl-2/adenovirus E1B 19-kDa interacting protein 3 (BNIP3), which is expressed upon T cell activation, inhibits BNIP3 degradation by the proteasome, thereby sensitizing T cells to apoptosis [26], [47].

At the end of the contraction phase, macrophages and neutrophils must eliminate unwanted (apoptotic or damaged) and “unfit” cells via phagocytosis [48]. CD47 serves as a “don’t eat me” signal, which inhibits cell clearance when delivered to SIRP-α^+^ cells [49]. Viable CD47^−/−^ T cells are quickly eliminated from a CD47^+/+^, but not CD47^−/−^ host, by SIRP-α^+^ cells [30], [31]. Notably, since CD47^−/−^ mice are viable, clearance of CD47^−/−^ cells does not occur in CD47^−/−^ mice because these SIRP-α^+^ macrophages must be educated by CD47 ^+/+^ stromal cells to acquire functional phagocytosis via interruption of the CD47/SIRP-α pathway [50]. Nonetheless, the contraction of CD4^+^CD44^hi^CD47^low^ T cells occurred in the immunized CD47^−/−^ host. In fact, a CD47^low^ status does not equate to absence of CD47. Of note, only 10% to 20% of normal CD47 expression on RBC is sufficient to prevent cell clearance [51]. Furthermore, Weissman and others demonstrated that concealing CD47 with antibodies on live cells is necessary but insufficient to trigger phagocytosis *in vivo*, since phagocytosis required the expression of calreticulin, which is upregulated on malignant cells [52]. In fact, the “turning off” of non-phagocytic signals must be coupled to the “switching on” of phagocytic signals to provoke cell elimination [48]. Among others, calreticulin serves as a pro-phagocytic signal, which, through binding to its macrophage counter-receptor low-density lipoprotein–related protein (LRP), leads to engulfment of the target cell [28]. In the present study, calreticulin expression was not detected on viable human memory CD4 T cell subsets. In contrast, killing by 4N1K peptide induced calreticulin expression on T_EM_ dying cells, indicating that CD47-mediated cell death represents an upstream event to the elimination of unwanted cells. CD47 expression/redistribution on apoptotic cells also appear to augment phagocytosis [28], [53].

Taken all, we present a key role for CD47 on CD4 T cells in the resolution of an inflammatory response and propose that the following sequence of events accounts for the elimination of a large number of effector T cells. Ag encounter induces a CD47^low^ status on TCR-activated CD4 T cells. Unless rescued by IL-2, which reverses their phenotype to CD47^high^ status, the majority of CD47^low^ T cells will become susceptible to killing by TSP-1 and then augment their expression of pro-phagocytic signals to promote their clearance by SIRP-α^+^ cells. Further studies that permit to modulate CD47’s status and T cell death may provide novel strategies for improved vaccination and/or the elimination of unwanted, auto-aggressive T cells in inflamed tissues such as in CD.

## Materials and Methods

### Ethics Statement

All mouse experimental protocols were approved by “Comité institutionnel de protection des animaux (CIPA) du Centre de recherche du Centre hospitalier de l’Université de Montréal (CRCHUM)” that follows the guidelines of the Canadian Council on Animal Care (CCAC). All the experiments were approved by “Comité d’éthique de la recherche du Centre hospitalier de l’Université de Montréal (CHUM)” and written informed consent was obtained from all donors. Human samples were obtained from healthy volunteers, umbilical cord blood and the patients recruited from the Gastroenterology and Surgery Division at CHUM.

### Clinical Samples

Peripheral blood samples were collected from all donors, and tissue samples were obtained from endoscopic biopsies or surgically resected specimens. Intestinal tissue samples were taken from unaffected areas of donors with non inflammatory bowel diseases (non IBD) or inflamed regions of Crohn’s disease (CD) patients. Mesenteric lymph nodes (mLNs) were collected after surgery by the pathologists.

### Animals

CD47^−/−^129sv/eg mice were backcrossed onto CD47^+/+^ BALB/c mice for 16 to 18 generations. Mice expressing the DO11.10 TCR transgene, which is specific for the peptide residues 323–339 of chicken OVA, were purchased from Charles River Laboratories and backcrossed into CD47^−/−^ mice. Female mice 6 to 10 weeks old were used in all experimental protocols and were maintained under specific pathogen-free conditions.

### Isolation of Cells

Peripheral blood mononuclear cells (PBMC) or umbilical cord blood mononuclear cells (CBMC) were prepared by density gradient centrifugation of heparinized peripheral blood. Lamina propria mononuclear cells (LPMC) were prepared from intestinal specimens using a modified protocol described by Bull and Bookman (1977). Briefly, the dissected mucosal tissue was cut into small pieces, incubated in HBSS (Sigma) with 1 mM DTT (Sigma) and 1 mM EDTA (Sigma) for 45 min at 37°C, followed by enzymatic digestion with 0.25 mg/ml of collagenase D (Roche) and 0.01 mg/ml of DNase I (Roche) for 45–60 min at 37°C, combined with mechanical dissociation by Dissociator (Miltenyi Biotech). Mesenteric LNs were harvested and squeezed on a 70 mm pore mesh to obtain a cellular suspension.

### Antibodies and Reagents

All the antibodies were purchased from Biolegend (USA) unless otherwise indicated. 2D3 cell line was obtained from Dr. E. Brown (Genentech, USA). Monoclonal antibodies against the following human antigens were used for labeling and sorting: CD45 (HI30) CD4 (RPA-T4), CD45RA (HI100), CD62L (DREG-56), CCR7 (TG8/CCR7), CD47 (B6H12 and 2D3), CD27 (MT271, BD) CCR5 (2D7/CCR5), CD127 (HIL-7R-M21) and Calreticulin (FMC 75, Assay designs). Antibodies against mouse antigens: CD4 (RM4–5), TCR (DO11.10) (KJ126). Human and mouse CD47 expression was also revealed using huSIRP-α-Fc and muSIRP-α-Fc fusion proteins that contain SIRP-αD1D2D3 domains fused to mutated human Fc IgG (Novartis, Basel, Switzerland), respectively [54]. For *in vitro* human T cell stimulation, anti-CD3 (OKT3, Janssen-Ortho) and anti-CD28 (CD28.2) mAbs were used.

### Flow Cytometry for Phenotypic Analysis

CD47 expression was examined with huSIRP-α-Fc or with anti-CD47 mAbs after gating on naive (T_N_: CD4^+^CD45RA^+^CCR7^+^), effector memory (T_EM_: CD4^+^CD45RA^−^CCR7^−^CD27^−^), and central memory (T_CM_: CD4^+^CD45RA^−^CCR7^+^CD27^+^) T cells. Staining was performed in FACS buffer (PBS supplemented with 2% FCS, 2 mM EDTA, and 0.01% sodium azide at 4°C for 30 min).

### Cell Culture

T_N_ and T_CM_ cells were isolated from PBMC or CBMC using a FACS Aria II sorter (BD). Purity was more than 99%. 1×10^6^ CFSE-labeled T_N_ or T_CM_ cells were stimulated in RPMI (Wisent Inc.) supplemented with 10% fetal calf serum (Wisent Inc), 500 U/ml penicillin, and 500 ug/ml streptomycin with immobilized anti-CD3 (10 ug/ml) and soluble anti-CD28 (2 ug/ml) mAbs for 6 days in 24-well plates (Costar). For secondary cultures, 0.5×10^6^ activated T_N_ cells were restimulated with coated anti-CD3 (10 ug/ml) and soluble anti-CD28 (2 ug/ml) with/without IL-2 (100 U, R&D system) or expanded only in IL-2 for 5 days in 48-well plates (Costar). For some experiments, CFSE-labeled activated CD4 T cells were stained with huSIRP-α-Fc protein and FACS-sorted according to CD47 status before restimulation.

### Protein Extractions and Immunoblot

Whole-cell extracts were prepared in 20 mM Tris-HCl pH 7.4, 150 mM NaCl, 1% Triton X-100, 10% glycerol, 2 mM EDTA, and antiprotease mixture (Roche). Protein content was determined with the Bio-Rad DC kit and 10 dodecyl sulfate polyacrylamide gel electrophoresis (SDS-PAGE). After blotting, Nitrocellulose filters were probed with 2D3 mAb and anti β-actin. Both were detected according to standard procedures.

### TSP-1 Production by Human Colonic Specimens

TSP-1 concentration (ng/ml) was measured with ELISA kit (Chemicon International) in human colon tissue lysates after homogenization and normalized per milligram of tissue.

### Confocal Microscopy

T_N_, T_EM_ and T_CM_ were FACs sorted from PBMC (purity >99.9%), stained with SIRP-α-Fc or anti-CD47 (B6H12) biotinylated and followed by streptavidin Dylight 649 (Biolegend). Samples were mounted in ProLong Gold (Invitrogen) and analyzed with a confocal microscope (Leica).

### CD47-mediated Killing Assays

Purified T cells (4×10^5^) isolated from PBMC were labeled with antibodies for CD4 T cell subsets before incubating with 200 uM of TSP-1-specific (4N1K, KRFYVVMWKK) or control (4NGG, KRFYGGMWKK) peptides (McGill University/Sheldon Biotechnology, Montreal) for 30 min at 37°C. Apoptotic cells were revealed with Annexin V binding (BD Bioscience) after gating on T_N_, T_EM_, and T_CM_ T cells. For some experiments, CFSE-labeled T_N_ were activated for 6 days and then FACS-sorted according to CFSE^low^ before *in vitro* secondary stimulation. After 5 days, CD47-mediated killing assays were performed under the same conditions as in *ex vivo* studies. Specific CD47-mediated killing was calculated as follows: percentage of Annexin^+^ cells in 4N1K-stimulated T cells, minus the percentage of Annexin^+^ cells in 4NGG-stimulated T cells.

### Adoptive Transfer Experiments

BALB/c CD47^+/+^ or CD47^−/−^ mice were passively transferred (intravenously, i.v.) with 2×10^6^ CFSE-labeled CD4 T cells isolated from CD47^+/+^ or CD47^−/−^ DO11.10 (Tg) mice. After 1 day, mice were immunized either subcutaneously (s.c.) with OVA protein (100ug/ml, Sigma) emulsified in complete Freund’sFreud adjuvant (MP Biomedicals, LLC) or anti-DEC205 coupled with OVA peptide (gift from R. Steinman, Rockefeller University). The phenotype of Tg T cells was analyzed by flow cytometry at different time points in draining LNs.

### Statistical Analysis

Statistical analyses were performed using the unpaired Student t test. One-way Anova test was used to compare the variation of CD47 expression between human CD4 T cell subpopulations. Data represent mean ± standard deviation (SD). (***P<.001; **P<.01; *P<.05).

## Supporting Information

Figure S1 JinB8, a CD47-negative Jurkat cell line, was transfected with various cDNA constructs of CD47 as previously described [12]. Cell lines were stained with anti-CD47 (B6H12 or 2D3) mAbs or huSIRP-α-Fc protein. Data are representative of 2 independent experiments.
(TIF)
