# Supplementary figures and images for: CD47Low Status on CD4 Effectors Is Necessary for the Contraction/Resolution of the Immune Response in Humans and Mice
Source: PLoS One. 2012 Aug 1;7(8):e41972. doi: 10.1371/journal.pone.0041972 (PMC3411572; doi:10.1371/journal.pone.0041972)

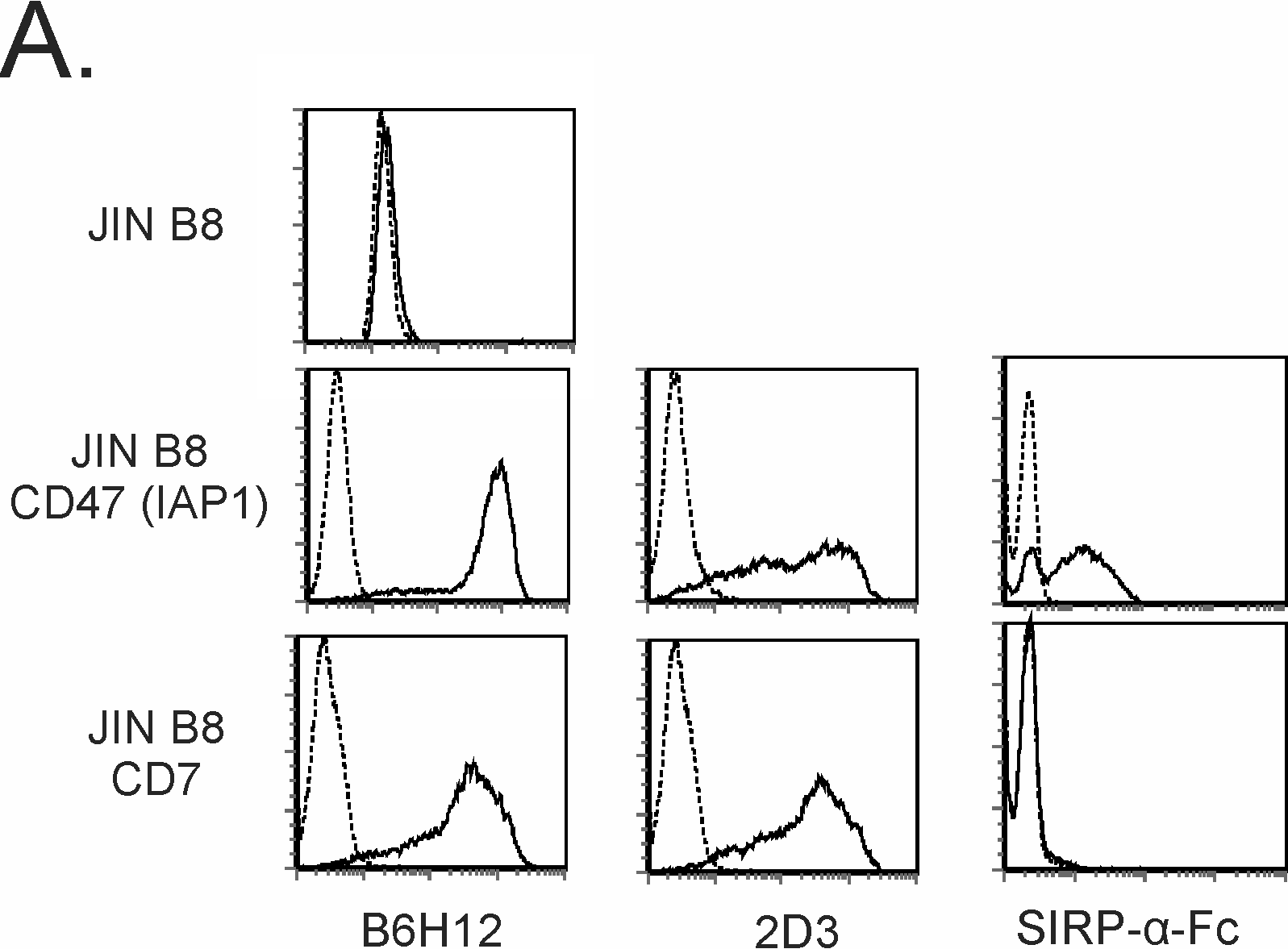

Supplement: Figure S1 — JinB8, a CD47-negative Jurkat cell line, was transfected with various cDNA constructs of CD47 as previously described [12]. Cell lines were stained with anti-CD47 (B6H12 or 2D3) mAbs or huSIRP-α-Fc protein. Data are representative of 2 independent experiments. (TIF) [file pone.0041972.s001.tif]
